# Lessons Learned in Practice with Li-Fraumeni Syndrome: LFS-Related Breast Cancer Treatment Strategy and Establishment of a Surveillance System

**DOI:** 10.14789/jmj.JMJ22-0012-CR

**Published:** 2022-08-02

**Authors:** RITSUKO SASAKI, YOSHIYA HORIMOTO, HARUMI SAEKI, SHOJI SATO, KATSUHIRO SANO, NAOTO SHIKAMA, MAYUMI UENO, MITSUE SAITO, MASAMI ARAI

**Affiliations:** 1 Department of Breast Oncology, Juntendo University Graduate School of Medicine, Tokyo, Japan; 1 Department of Breast Oncology, Juntendo University Graduate School of Medicine, Tokyo, Japan; 2 Department of Human Pathology, Juntendo University Graduate School of Medicine, Tokyo, Japan; 2 Department of Human Pathology, Juntendo University Graduate School of Medicine, Tokyo, Japan; 3 Department of Radiology, Juntendo University School of Medicine, Tokyo, Japan; 3 Department of Radiology, Juntendo University School of Medicine, Tokyo, Japan; 4 Ogurihara Clinic, Masamakai Medical Corporation, Tokyo, Japan; 4 Ogurihara Clinic, Masamakai Medical Corporation, Tokyo, Japan; 5 Department of Clinical Genetics, Juntendo University Graduate School of Medicine, Tokyo, Japan; 5 Department of Clinical Genetics, Juntendo University Graduate School of Medicine, Tokyo, Japan

**Keywords:** Li-Fraumeni syndrome, hereditary cancer syndrome, treatment management, surveillance, whole-body MRI

## Abstract

We herein present the case of a 33-year-old woman with no family history of metachronous bilateral breast cancer and osteosarcoma, diagnosed with Li-Fraumeni syndrome (LFS), which is a rare autosomal dominant hereditary cancer syndrome associated with a germline *TP53* variant. She was diagnosed with left distal femoral osteosarcoma at the age of 16, and metachronous bilateral breast cancer at the ages of 29 and 33. When the third cancer was diagnosed, a hereditary tumor syndrome was suspected and the patient was referred to our genetic outpatient clinic. There was no family history of the ‘core’ cancers for LFS, but since the patient met Chompret’s criteria, germline *TP53* genetic testing was performed with the patient’s will. A pathogenic variant, *TP53*:c.216dupC (p.Val73ArgfsX76) was found in exon 4 of the gene. This case is didactic because radiotherapy was performed on the first breast cancer before the diagnosis of LFS was made; radiation should be avoided if there are other options in LFS because of the inability to repair DNA damage. As a lesson learned, oncologists reaffirmed the importance of being aware of hereditary tumors from the keywords “multiple,” “young,” “familial,” and “rare,” and consulting the genetic department. In addition, surveillance using whole-body magnetic resonance imaging is recommended in LFS. However, this system is not yet provided nationwide, but we have newly settled it in our hospital.

## Introduction

Li-Fraumeni syndrome (LFS) is a rare autosomal dominant hereditary cancer syndrome associated with germline *TP53* pathogenic or likely pathogenic variants^1)^. The tumor suppressor gene, *TP53*, is located on chromosome 17, and the protein product of *TP53* is localized in a cell nucleus and binds directly to DNA. It has been called the “guardian of the genome” and plays important roles in controlling the cell cycle and apoptosis^2)^. The frequency of germline *TP53* variants in the general population has been reported to be about 1.6% in pediatric cancer patients^3, 4)^ and about 0.2% in adult cancer patients^5)^. The penetrance of germline *TP53* variants is 75% in males and almost 100% in females^6)^. Regarding age of cancer onset, an analysis of 415 individuals with *TP53* pathogenic variant in 214 French families with LFS showed that the cancer penetrance for young people aged 0, 5, and 18 years were 4%, 22%, and 41%, respectively^6)^. Furthermore, an analysis by the US National Cancer Institute of 286 *TP53* pathogenic variants in 107 families with LFS showed that the 50% cumulative cancer onset age was 46 years for male and 31 years for female^7)^. It has been reported that 12.2 % of cases are de novo LFS; therefore, cases without a family history are scattered^8)^. LFS has a wide tumor spectrum, including the so-called ‘core' LFS cancers: soft-tissue sarcomas, osteosarcomas, adrenocortical carcinomas, central nervous system tumors, and very-early onset female breast cancer^9)^. Juvenile onset of cancer and multiple onsets and types of cancers in one patient are characteristics of LFS. Clinical guidelines for the management of LFS have been published in various countries, including Japan^10)^.

According to a report on the analysis of hereditary breast cancer-related genes in Japanese breast cancer patients, the percentage of patients with germline *TP53* variants is 3.9%, which is small compared to 72.5% for germline *BRCA1/2* variants^11)^. Therefore, LFS is rarely encountered in breast cancer treatment, and it is not easy to make a diagnosis. However, in the treatment of breast cancer, radiation therapy (RTx) is often used concomitantly, and the relative contraindication in this disease should be taken into consideration when making treatment decisions^12)^. This report describes a case of LFS that was suspected by its history and clinical course, despite no LFS-related family history of hereditary tumors, which led to LFS genetic testing.

## Case report

**Figure 1:**
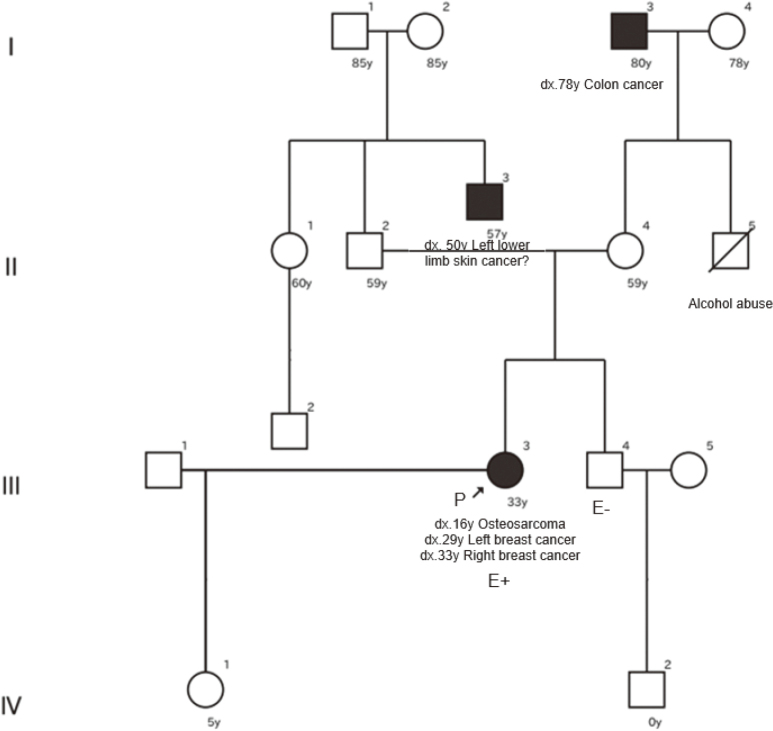
Family history Dx, age at diagnosis; d, age at death; y, years old.

A 33-year-old woman was diagnosed with distal left femoral osteosarcoma at the age of 16 due to pain in the left knee. She received perioperative chemotherapy and local excision and has had no signs of recurrence until now. At the age of 29, a left breast mass was detected by ultrasound screening and diagnosed as breast cancer. She underwent breast-conserving surgery and sentinel node biopsy, and was diagnosed with pT1bN0M0, Stage I, invasive micropapillary carcinoma (5×4 mm; estrogen receptor [ER] 60%, progesterone receptor [PgR] 90%, human epidermal growth factor receptor 2 [HER2] 3+, Ki67 70%; surgical margin positive at the lateral margin for in situ component). She received weekly paclitaxel+trastuzumab, LH-RH agonist, tamoxifen and RTx as postoperative therapy. Four years after the surgery for left breast cancer, at the age of 33, calcification of her right breast was detected on mammography. A stereotactic core biopsy was performed and diagnosed as breast cancer, cTisN0M0 Stage 0 (ductal carcinoma in situ [DCIS]). When she was diagnosed with metachronous contralateral breast cancer, a hereditary tumor was suspected and she was referred from her family clinic to our genetic department. Her family history included a maternal grandfather with colorectal cancer at the age of 78 and a paternal uncle with lower leg suspected skin cancer at 50 years (Figure 1). This patient had no obvious LFS-related family history. However, since the patient met Chompret's criteria (2015, Table 1) with multiple cancers and early-onset breast cancer in the ‘core' tumors, we performed germline *TP53* genetic testing (FALCO Ltd., Kyoto, Japan), with her consent (written informed consent was obtained from the patient.). The genetic test results revealed a pathogenic variant, *TP53*:c.216dupC (p.Val73ArgfsX76) was found in exon 4 of the gene, and a diagnosis of LFS.

**Table 1:** 2015 version of Chompret's criteria for Li-Fraumeni syndrome diagnosis

Familial presentation	Proband with TP53 core cancer before 46 years and at least one first- or second-degree relative with a core tumor before 56 years.
Multiple primitive tumors	Proband with multiple tumors, including two TP53 core tumors, the first of which occurred before 46 years, irrespective of family history.
Rare tumors	Patient with adrenocortical carcinoma, choroid plexus carcinoma or rhabdomyosarcoma of embryonal anaplastic subtype, irrespective of family history.
Very early-onset breast cancer	Breast cancer before 31 years, irrespective of family history.

**Figure 2:**
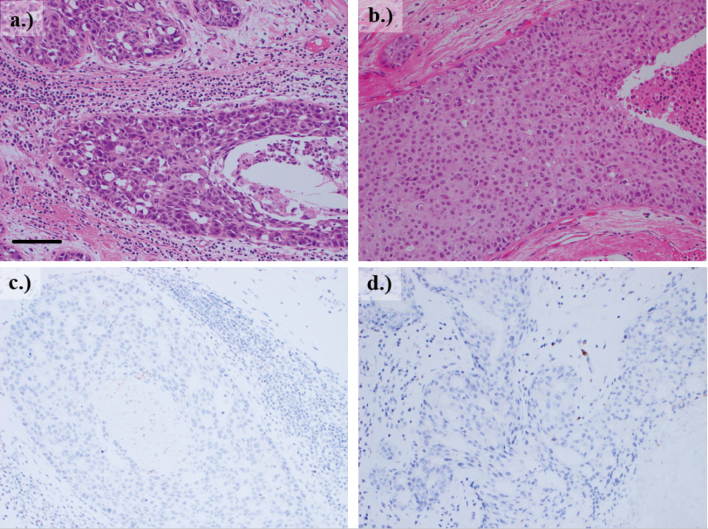
Pathological features of the surgical specimens a) and c) right breast cancer; b) and d) left breast cancer. a-b) hematoxylin and eosin staining ×100; b-d) p53 staining x100.

The patient was referred to the department of breast surgery through the genetic department for further treatment. At the same time as the right mastectomy, the patient preferred to have a left residual mastectomy because of the positive DCIS margins at the previous surgery and for risk reduction. She underwent bilateral mastectomy and breast reconstruction. The postoperative pathology results were as follows. The right breast cancer was DCIS solid > cribriform type, 3×2×12 mm, NG2, pTisN0M0, Stage 0 (ER-, PgR-, HER2 3+, Ki67 40%). The left breast was assessed in every 1 cm-slice for the entire section, due to the residual mastectomy being for risk reduction. Close to the previous surgical scar, DCIS was found (tumor size: 12×6×20 mm, comedo type, NG2, pTisNxMx), which was considered to be a residual lesion due to its similar histology. Additionally, p53 immunohistochemical staining (monoclonal, anti-mouse, clone PAb1801) was negative (Figure 2). Sporadic breast cancer cases in the control group that were in situ breast cancer and of similar subtype also tested negative.

**Figure 3:**
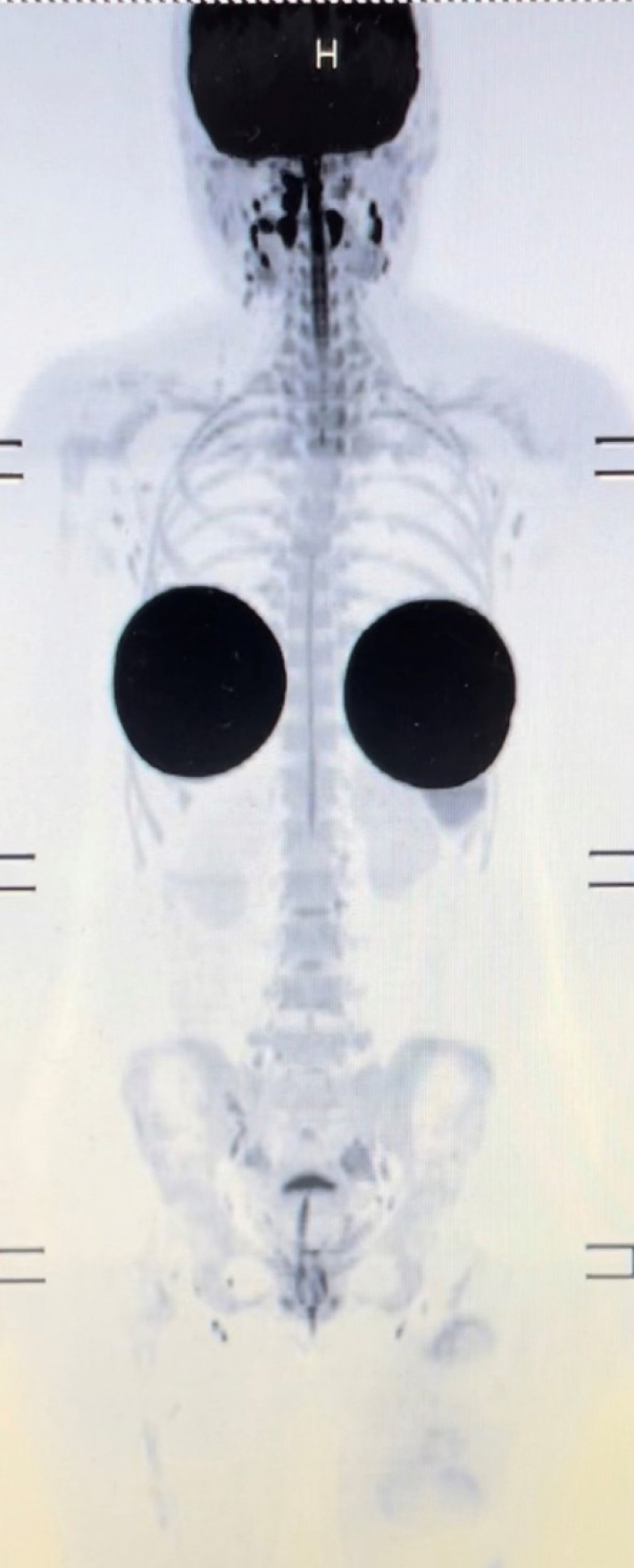
Representative image of whole-body magnetic resonance imaging surveillance

**Figure 4:**
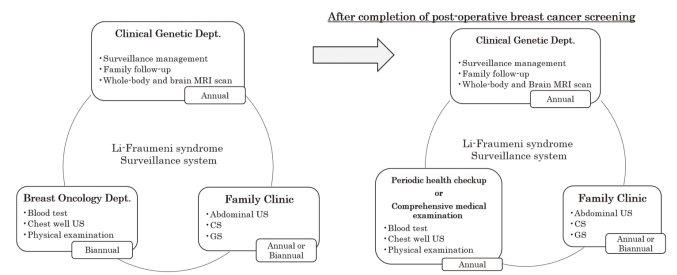
Conceptualization of a long-term Li-Fraumeni syndrome surveillance system to be conducted in collaboration among departments. US, ultrasound; CS, colonoscopy; GS, gastroscopy

Currently, she is receiving endocrine therapy for left breast cancer and will be followed up in our department for 5 years after surgery, including blood tests and chest wall ultrasound. In contrast, LFS requires lifetime cancer surveillance. Since the surveillance for osteosarcoma has been performed by semi-annual chest and local X-rays, we decided to introduce a surveillance program according to the Toronto protocol^13)^, which avoids radiation exposure and follow-up drop outs. Through this case, we have just settled a system of annual whole-body magnetic resonance imaging (MRI) examinations in cooperation with the radiology department (Figure 3). In addition, we decided to conduct colonoscopy and abdominal ultrasound every one to two years in cooperation with her family clinic (Figure 4).

This case is speculated be *de novo* onset of LFS because there is no LFS-related family history and due to the high penetrance of the syndrome; the parents have no history of abnormal findings on annual examinations to date. The younger brother underwent carrier diagnosis and no variants were detected. Hereafter, the patient is also considering carrier diagnosis for her 5-year-old daughter, taking into account the advantages and disadvantages.

## Discussion

### Characteristics of LFS-related breast cancer

Breast cancer is the most common LFS-related tumor, accounting for 27% to 31% of all reported LFS tumors^6)^. LFS-related breast cancer is characterized by a median age as young as 33 years, and almost all cases occur before menopause. Among younger breast cancer patients under 30 years, germline *TP53* variants were detected in about 4%-8% of cases (without germline *BRCA1/2* pathogenic variants). There are few reports on clinicopathological characteristics; one report compared 30 LFS breast cancers with 79 sporadic cases and showed a significantly higher rate of HER2 positivity in the LFS group (67% vs. 25%, respectively; p<0.001)^14)^. In addition, 65% of LFS breast cancers were bilateral. Our case had similar pathological characteristics and clinical course.

### Radiation exposure in LFS

LFS-related breast cancer treatment strategies, such as radiation, surgery and drug therapy, are listed in Table 2 according to consensus recommendations^12)^. The *TP53* gene is the most important tumor suppressor gene in preventing cancer development. It plays an important role in cell cycle regulation and apoptosis by providing cells with the ability to respond to and repair DNA damage after cellular stress and by triggering multiple downstream repair pathways. Therefore, radiation exposure, which increases the risk of developing a second cancer, should be avoided if there are other options in LFS because DNA damage cannot be repaired. In the present case, LFS was not suspected at the time of the initial breast cancer, and treatment and surveillance with RTx and X-rays were performed. It is not easy for a non-geneticist to suspect a hereditary tumor, other than hereditary breast-ovarian cancer syndrome (HBOC). However, it may be necessary to suspect the possibility of a background hereditary tumor related to breast cancer, as it may affect treatment choices.

Meanwhile, there are cases where the benefits to the patient exceed the harms in cancer treatment. The decision of whether or not to prescribe RTx in LFS patients with cancer relies on a delicate multidisciplinary assessment of the risk of a second cancer (based on age and pathogenic variants, or whether heterozygous or in a state) and the oncological prognosis^15)^.

**Table 2:** Consensus recommendations for the management of Li-Fraumeni syndrome^12)^

Radiation therapy	RT of the intact breast is contraindicated.
	Postmastectomy RT should only be considered in patients with a significant risk of locoregional regional recurrence.
	〔Strength of recommendation: Moderate, Quality of evidence: Low (case-series only)〕
Surgical therapy	A mastectomy is the recommended therapeutic option.
Systemic drug therapy	Avoid cytotoxic anticancer drugs that induce DNA damage if possible. PARP inhibitors: insufficient evidence for moderately penetrant genes including TP53.
Diagnostic Imaging	Avoid radiation exposure (e.g.: ultrasound, MRI).

MRI, magnetic resonance imaging; RT, radiation therapy.

### Surgical treatment in LFS

Total mastectomy should be the surgical treatment of choice for LFS. As for risk-reducing strategies, in HBOC, contralateral risk-reducing mastectomy (CRRM) is covered by insurance. However, CRRM is not recommended for breast cancer patients who have a mutation in a moderate-penetrance breast cancer gene, and therefore an application to an ethics committee is required. As a rare disease, it is difficult to establish evidence regarding LFS, but we believe that it is necessary to provide medical care based on individual requests. In this case, the patient had a positive DCIS margin, which made it appropriate to perform a residual mastectomy^16)^. The local recurrence rate is twice as high when the margin of DCIS is positive as when it is negative, and half of the patients with DCIS recurrence will have invasive cancer, so complete resection is recommended.

Our case underwent reconstruction, and in recent years there have been increased warnings about breast-implant-associated anaplastic large cell lymphoma (BIA-ALCL), which is a rare form of T-cell lymphoma that occurs in some people who have had breast implants. BIA-ALCL in LFS has been accumulating and also requires attention^17)^. In this case, reconstruction was performed using a smooth type of implant, but there is a report recommending removal in cases of LFS using implants with textured surfaces. BIA-ALCL-derived cell line studies have shown evidence of dysregulation of p53 signaling pathways in response to DNA damage^18)^. Therefore, it is possible that BIA-ALCL develops due to inadequate tumor-suppressor activity in LFS.

### Characteristics of the variant in this case

In LFS, missense variants account for about 70% of cases, and the variant in this case is a frameshift variant that has been reported in three cases, two of them developed osteosarcoma, breast cancer and no evidence of onset in childhood (Table 3)^19, 20)^. Genotype-phenotype correlation has been reported for the *TP53* gene, and variable phenotypes, expressivity, and penetrance of cancer are frequent^21)^. Recently, variants leading to loss of function of p53 have been reported to have a more severe phenotype than patients with partial loss^22)^. The loss-of-function form has an early onset of primary cancer, often developing sarcoma or breast cancer by the age of 35 and meeting the classical LFS and Chompret's criteria. The pathological variant in this case has also been reported to show normal p53 protein loss of function. In this case, the frameshift mutation has altered the structure of the p53 protein itself, so it is not detected by immunostaining.

**Table 3:** Comparison between previous reports and this case with c.216dupC in *TP53* (p.Val73Arg fsX76)

	Togucihda et al 1992	Zerdoumi Y et al 2017	Our case 2021
Medical History	15yF Osteosarcoma	Pt22, 27yF Breast cancer Pt21, 28yM Unaffected	16yF Osteosarcoma 29y Lt-breast cancer 33y Rt-breast cancer
Family history	Mother: 25y Breast cancer	Not listed	Not specific

F, female; M, male; Lt, left; Rt, right; y, years old; Pt, patient.

The possibility of a paternal origin has not been completely ruled out, since the LFS has a 75% male penetrance and the paternal uncle is also suspected of having skin cancer. However, since the probability suggests that our case could be a single case, it is necessary to consider the possibility of germline mosaicism, which occurs at a frequency of 2.4% in addition to *de novo*^8)^.

### Establishing a continuous surveillance system

A lifetime risk of developing cancer, and regular systemic screening is considered important in LFS. Currently, surveillance based on the Toronto protocol has been introduced worldwide for *TP53* carriers (Table 4)^13)^. Meanwhile since there are some differences in surveillance subjects and the timing of the start of surveillance subject depending on the region, it is better to refer to the guideline by Kumamoto et al. in Japan^10)^. It is not clear whether the current surveillance studies contribute to improved survival rates due to the short observation period and the low incidence of the disease. However, in a meta-analysis of whole-body MRI surveillance, most of the cancers detected were localized and curative treatment was achieved^23)^. Therefore, there is a high possibility of reducing complications and improving quality of life by reducing the intensity of treatment. In addition, some studies have reported that routine examinations can contribute to a reduction in anxiety^24)^.

**Table 4:** Toronto protocol: agreed surveillance recommendations for *TP53* carriers^13)^

ACC	Abdominal US, every 3–4 month: birth-40 y, Biochemistry (17 OH-progesterone, total testosterone, DHEAS, androstenedione) should only be performed where there is an unsatisfactory USS.
Breast cancer	Annual dedicated MRI from age 20–70 years
	(woman only) Consider risk-reducing mastectomy from age 20 years
Brain tumor	Annual dedicated brain MRI from birth (first MRI with contrast)
Sarcoma	Annual WB-MRI from birth, Abdominal US 3-4 monthly: from 18 y
Colon	Colonoscopy every 2 y: from age 25 or 10 y before earliest onset of colorectal carcinoma in family
Gastric	Recommend Helicobacter pylori testing and eradication if required
	Endoscopy not indicated due to lack of evidence
Skin	Annual dermatology review from 18 years (general practitioner or dermatology), Japanese is not common. General advice on use of high protection factor sunscreen and covering up in sun.
Other	Recommend detailed discussion of red flag symptoms in both children and adults and provide information on relevant resources. Discuss importance of making positive lifestyle choices (e.g.: not smoking, eating a healthy diet, limiting alcohol consumption, sun protection, keeping physically active and providing appropriate resources).

ACC, adrenocortical carcinoma; DHEAS, dehydroepiandrosterone sulfate; MRI, magnetic resonance imaging; US, ultrasound; WB, whole-body; y years.

Due to the rarity of the disease, the annual surveillance system recommended for LFS is not currently in place nationwide. Although some other facilities perform brain, thoracic, abdominal, and pelvic (including lower limb) imaging four times a year, surveillance that requires frequent visits lacks continuity. Therefore, we proposed a surveillance system that includes whole-body and brain MRI^25)^, with collaborations between the genetics department, radiology department, breast oncology department and the family clinic.

## Conclusion

With the spread of cancer genomic medicine, the diagnosis rate of hereditary tumor syndromes is expected to increase in the future. For these diseases, extra consideration should be given when deciding on a cancer treatment plan and a lifetime surveillance system is necessary. At our hospital, which has a genetic outpatient clinic, we have established a surveillance system including whole-body MRI for LFS, which hopefully will be taken up by other hospitals in Japan.

## Funding

The author recived no financial support for the research.

## Author contributions

RS carried out the acquisition of data and wrote the manuscript. YH and HS conducted pathological assessments. KS and SS conducted whole-body MRI test and assessments. NS and MA reviewed and revised the manuscript. MU, MS, and MA were responsible for the care of the patient. All authors read and approved the final manuscript.

## Conflicts of interest statement

None declared.
